# Crystal structure reinvestigation and spectroscopic analysis of tricadmium orthophosphate

**DOI:** 10.1107/S2056989023009775

**Published:** 2023-11-14

**Authors:** Fatima-Zahra Cherif, Mhamed Taibi, Ali Boukhari, Abderrazzak Assani, Mohamed Saadi, Lahcen El Ammari

**Affiliations:** a Laboratoire de Chimie Appliquée des Matériaux, Centre des Sciences des Matériaux, Faculty of Science, Mohammed V University in Rabat, Avenue Ibn Batouta, BP 1014, Rabat, Morocco; b Laboratoire de Physico-Chimie des Matériaux Inorganiques et Organiques, Centre des Sciences des Matériaux, Ecole Normale Supérieure, Mohammed V University in Rabat, Morocco; Universidad de la República, Uruguay

**Keywords:** crystal structure, cadmium phosphate, orthophosphates, infrared spectroscopy

## Abstract

Single crystals of tricadmium orthophosphate, Cd_3_(PO_4_)_2_, have been synthesized successfully by the hydro­thermal route, while its powder form was obtained by a solid-solid process. The corresponding crystal structure was determined using X-ray diffraction data in the monoclinic space group *P*2_1_/*n*. The crystal structure consists of Cd_2_O_8_ or Cd_2_O_10_ dimers linked together by PO_4_ tetra­hedra through sharing vertices or edges. Scanning electron microscopy (SEM) was used to investigate the morphology and to confirm the chemical composition of the synthesized powder. Infrared analysis corroborates the presence of isolated phosphate tetra­hedrons in the structure. UV–Visible studies showed an absorbance peak at 289 nm and a band gap energy of 3.85 eV, as determined by the Kubelka–Munk model.

## Abstract 2

The transition-metal orthophosphate, β-Cd_3_(PO_4_)_2_, was synthesized by a solid-state reaction and characterized by single-crystal X-ray diffraction and EDS spectroscopy. It crystallizes in the monoclinic system, space group *P*2_1_/*n*.

## 1. Chemical context

Phosphates with transition metals have received significant attention due to their wide range of potential applications in different fields of technology such as farming, energy storage, and in the medical field, as medicines or for diagnosis. For instance, Ni_3_(PO_4_)_2_ was identified as a heat-sensitive pigment and a catalyst for breaking and de­hydrogenating aliphatic hydro­carbons (Correcher *et al.*, 2013 ▸), while the orthophosphates Zn_3_(PO_4_)_2_ and Cu_3_(PO_4_)_2_ have been applied in dentistry as a component of tooth fillings and environmental contamination control (Servais & Cartz, 1971 ▸ and Rong *et al.* 2017 ▸), respectively. The aim of this paper is to provide a comprehensive discussion on the crystallographic arrangement of the β-Cd_3_(PO_4_)_2_ structure and to supply a full crystallographic description. Additionally, we will present the findings of our investigations into the compound’s optical and morphological properties.

## 2. Structural commentary

**Structural study**

On the basis of the single crystal X-ray diffraction data analysis, the tri-cadmium orthophosphate crystallizes in the monoclinic system, space group *P*2_1_/*n*. In this phosphate structure, all cadmium, phospho­rus and oxygen atoms occupy the general Wyckoff positions 4*e*. The anisotropic refinement of all atoms belonging to the crystal structure of β-Cd_3_(PO_4_)_2_ leads to excellent merit factors {*R*[*F^2^* > 2σ(*F^2^*)] *=* 0.023, *wR*(*F^2^*) *=* 0.054 and *S* = 1.07}, which corroborate the adopted crystallographic model.

**Structural description**

The crystal structure of β-Cd_3_(PO_4_)_2_ presents a 3D framework constructed from isolated PO_4_ tetra­hedra and two different types of cadmium polyhedra, of coordination numbers five and six. The cadmium polyhedra are linked together to form a 3D framework. In this crystal structure, six of the nine cadmium atoms (Cd1, Cd2, Cd3, Cd4, Cd5, and Cd6) are located inside five-vertex polyhedra, with Cd—O bond lengths ranging from 2.1454 (19) to 2.3996 (18) Å, and averaging 2.2613 Å. The remaining three cadmium atoms (Cd7, Cd8, and Cd9) are located at the centers of six vertex polyhedra, with Cd—O bond lengths ranging from 2.2156 (17) to 2.5935 (19) Å, and averaging 2.3282 Å. The nearly regular phosphate tetra­hedra in the structure have P—O bond lengths ranging from 1.527 (2) to 1.557 (2) Å, and averaging 1.538 Å. On the other hand, while Stephens (1967 ▸) noted irregularities in the PO_4_ tetra­hedra due to incomplete refinement of the structure, in this case the structure has been fully refined and the PO_4_ tetra­hedra are all regular as shown by the bond lengths and the inter­atomic angles recorded in the supporting information.

The calculated bond-valence-sum (BVS) values (Brown & Altermatt, 1985 ▸) of all atoms in the crystal structure are in good agreement with the expected valence states of +5 for each of the six phospho­rus atoms, +2 for each of the nine cadmium atoms. and almost −2 for all oxygen atoms except O6 (BVS −1.669). Nevertheless, we see that all oxygen atoms are each linked to one phospho­rus atom and two cadmium atoms except O6, which is linked to one phospho­rus atom and a single cadmium atom. In this context, if we take into account the contribution of the two cadmiums located at distances of 2.729 and 2.731 Å, the BVS (O6) will be equal to −1.884. This indicates that the structure is nearly ionic, with each ion donating or accepting the expected number of valence electrons.

**Figure 1:**
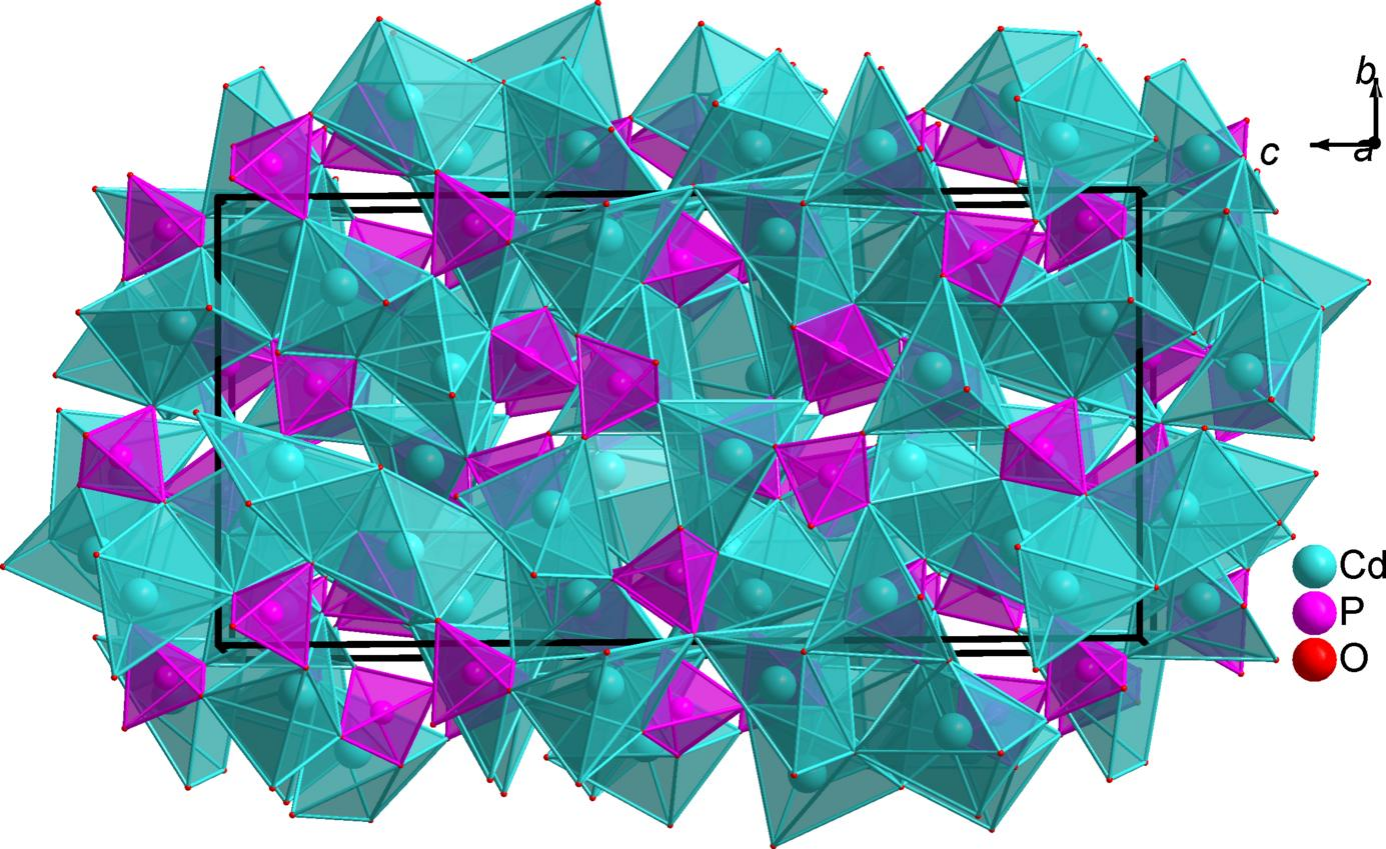
Three-dimensional view the β-Cd_3_(PO_4_)_2_ crystal structure showing small tunnels along the *a-*axis direction.

**Figure 2:**
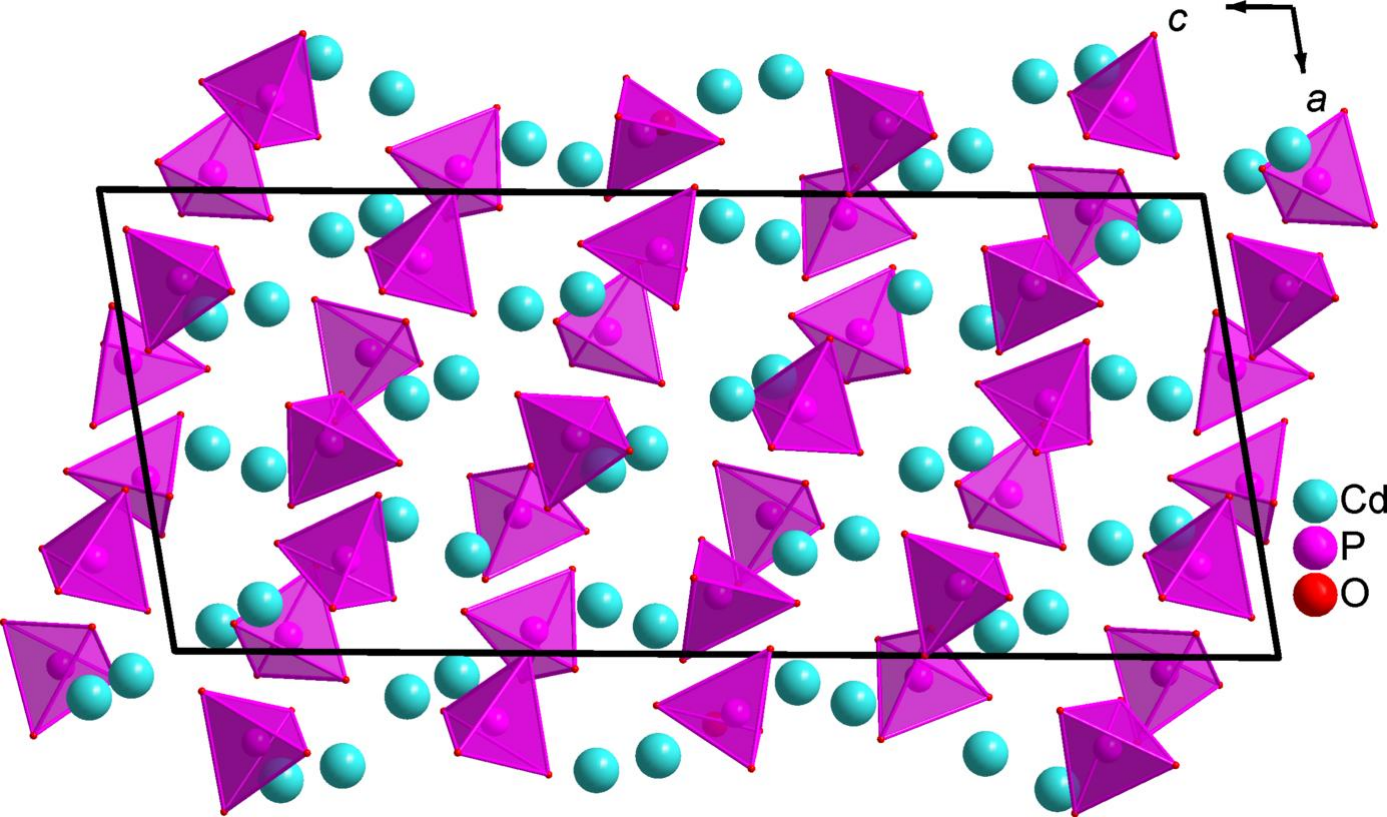
The anionic network of β-Cd_3_(PO_4_)_2_, formed by layers of PO_4_ tetra­hedra stacked along the [ 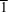 01] direction.

**Figure 3:**
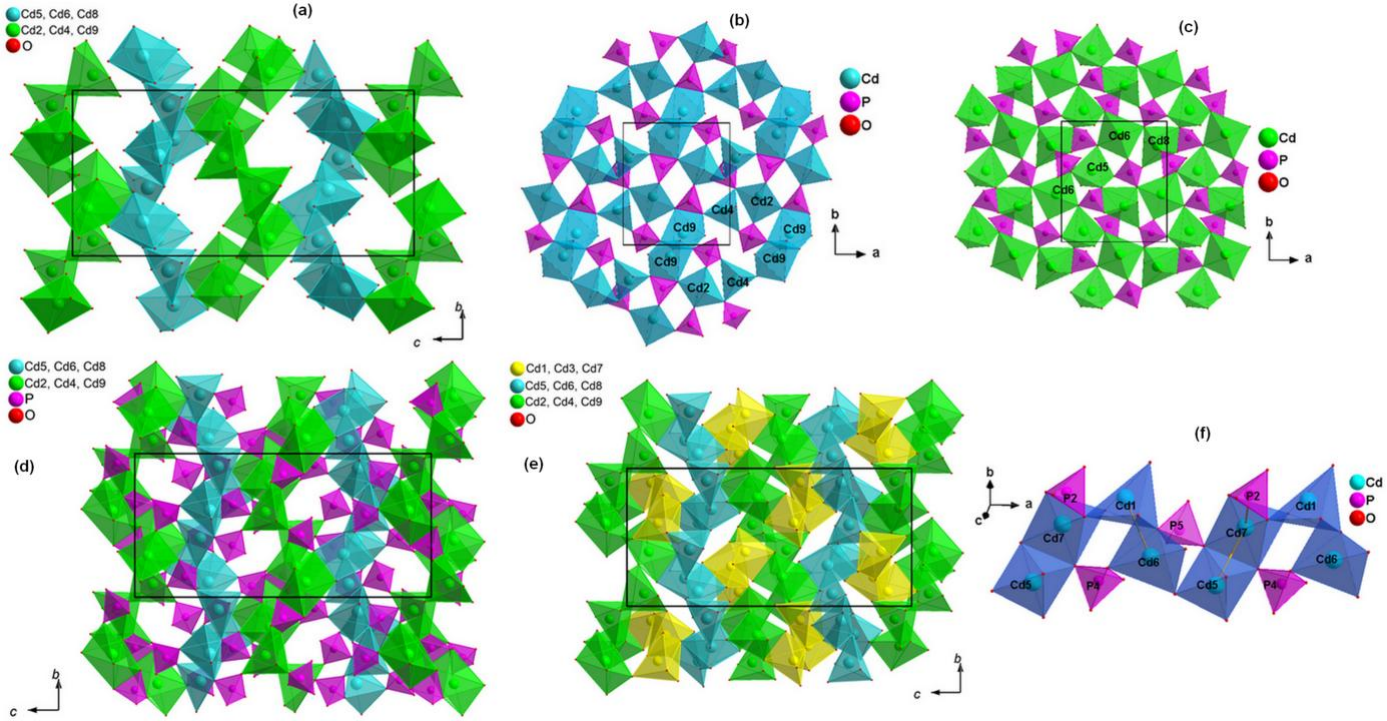
Schematic representation of the three dimensional coordination of the β-Cd_3_(PO_4_)_2_ structure; (*a*) two successive layers parallel to the *ab* plane of the cations (Cd2, Cd4 and Cd9) and (Cd5, Cd6 and Cd8), (*b*) Cd2O_5_, Cd4O_5_ and Cd9O_6_ polyhedra forming a ring of eight polyhedra, (*c*) two pyramids (Cd5O_5_, Cd6O_5_) and a deformed octa­hedron (Cd8O_6_) arranged to share corners building the second layer, (*d*) first and second layer connected, (*e*) a zigzag chain composed of the two pyramids Cd1O_5_ and Cd3O_5_ and the Cd7O_6_ prism fills the large tunnels of the framework, (*f*) the sequence of the three pyramids Cd1O_5_, Cd5O_5_, Cd6O_5_ and the square-based prism Cd7O_6_.

This phosphate has a complex crystal structure composed of almost regular PO_4_ tetra­hedra linked to two different types of distorted cadmium polyhedra arranged in a specific pattern to form a three-dimensional framework with small tunnels along the *a*-axis direction (see Fig. 1 ▸). Moreover, the anionic network is formed by layers of PO_4_ tetra­hedra stacked nearly along the [ 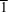 01] direction and the cadmium polyhedra fill the remaining space as shown in Fig. 2 ▸. Furthermore, the description of the sequence of the cadmium polyhedra is not easy. Indeed the polyhedra surrounding the cations (Cd2, Cd4 and Cd9) and (Cd5, Cd6 and Cd8) form two successive layers parallel to the *ab* plane (Fig. 3 ▸ *a*). The first layer of the framework consists of Cd2O_5_, Cd4O_5_ and Cd9O_6_ polyhedra, forming a ring of eight polyhedra two pairs of square-based prisms that share an edge and four pyramids linked by the vertices (Fig. 3 ▸ *b*). The second layer is composed of two Cd5O_5_, Cd6O_5_ pyramids and a deformed Cd8O_6_ octa­hedron arranged to share corners (Fig. 3 ▸ *c*). This layer is connected to the first layer to form a 3D framework with tunnels along the *a*-axis direction (Fig. 3 ▸ *d*). The zigzag chain composed of the remaining cadmium polyhedra, namely two pyramids, Cd1O_5_, Cd3O_5_, and the Cd7O_6_ prism fills the large tunnels of the framework (Fig. 3 ▸ *e*). The polyhedra belonging to this chain share the edges or vertices and form a zigzag pattern in the tunnels, which consolidates the connection of the two layers. Moreover, a more laborious examination of the structure shows that the four groups of cadmium polyhedra Cd5O_5_–Cd7O_6_–Cd1O_5_–Cd6O_5_ share edges to form slabs, which are linked together by the corners to build an infinite zigzag chain along the *a*-axis direction, as shown in Fig. 3 ▸ *f*.

**Powder X-ray diffraction**

**Figure 4:**
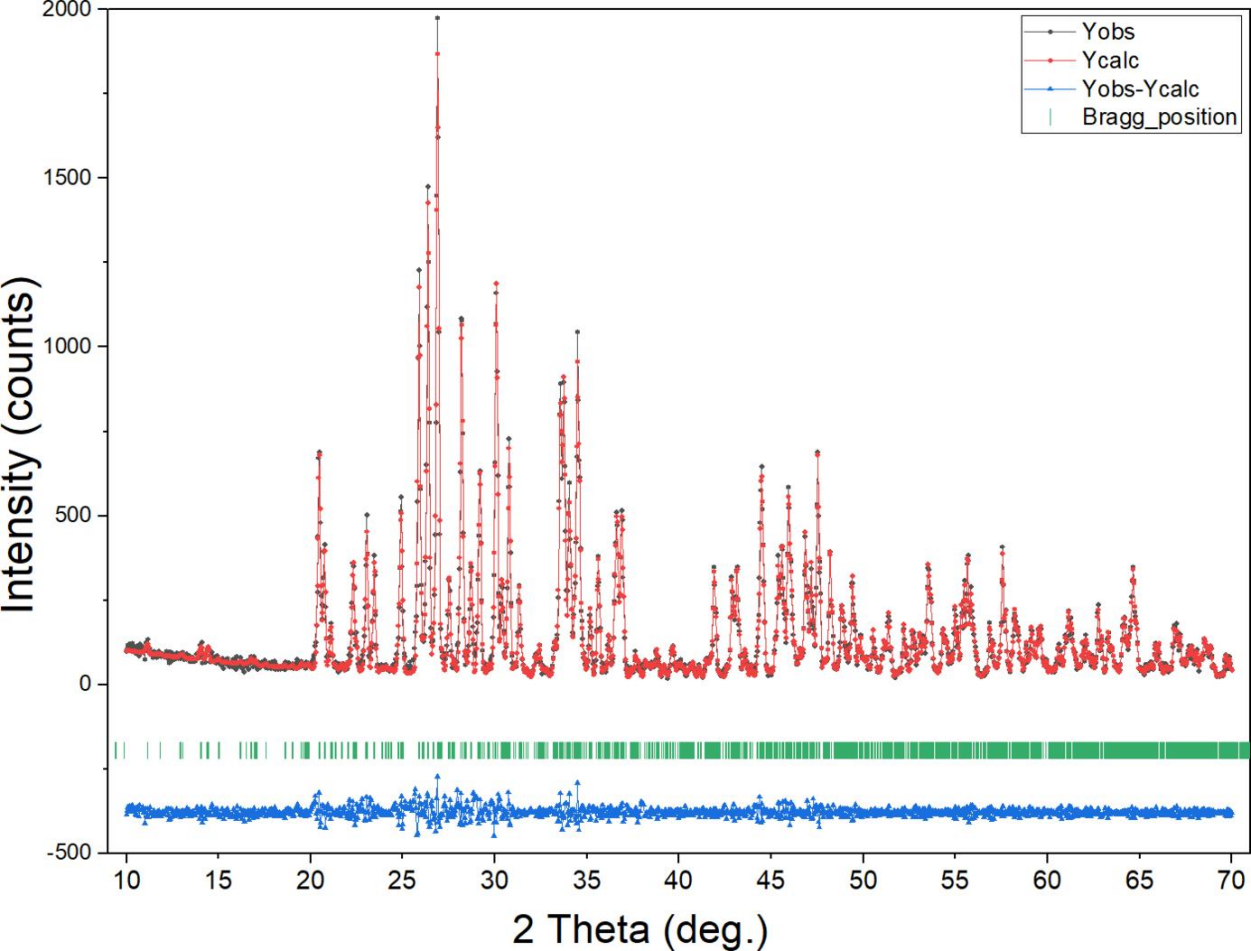
Calculated and observed X-ray diffraction patterns for β-Cd_3_(PO_4_)_2_.

The single crystal diffraction analysis and refinement of β-Cd_3_(PO_4_)_2_ produced high-quality crystallographic data, which were then used to run a profile matching with a Le Bail approach for X-ray powder analysis. This study leads to a very good match (Fig. 4 ▸), confirming the unit-cell parameters and space-group symmetry of the compound. The obtained lattice parameters are *a* = 9.1861 (8) Å, *b* = 10.3349 (8) Å, *c* = 21.689 (2) Å, and β = 99.575 (3)°, in the monoclinic system, space group *P*2_1_/*n*. This fact is corroborated by the good merit factors: *R*_p_ = 8.1%, *R*_wp_ = 11.7%, *R*_exp _ = 8.5%, χ2 = 1.904.

**Fourier-transform infrared analysis**

**Figure 5:**
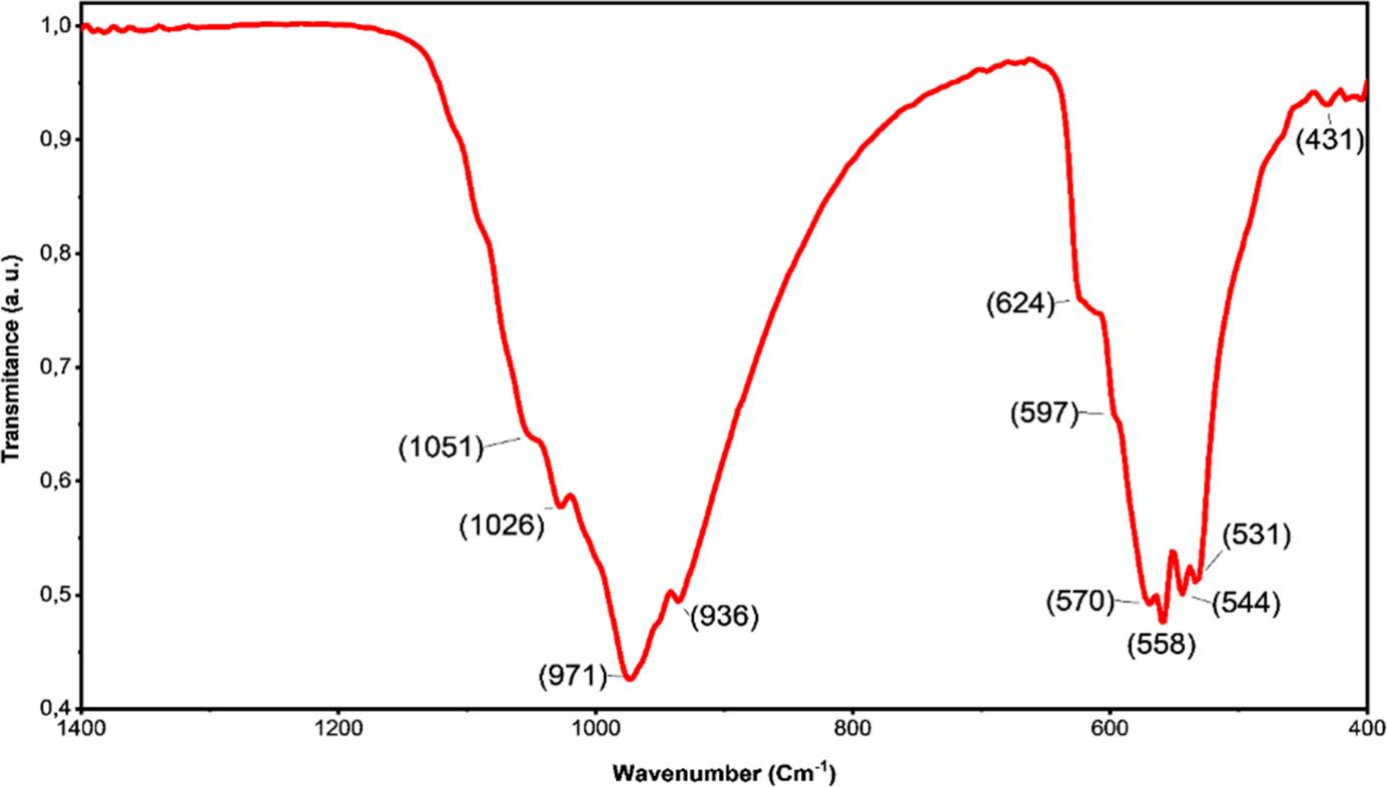
FT–IR spectra of β-Cd_3_(PO_4_)_2_.

Fig. 5 ▸ presents the FTIR spectra of β-Cd_3_(PO_4_)_2_, displaying two distinct regions of bands that originate from the [PO_4_]^3−^ groups. The first region, ranging from 1151 to 936 cm^−1^, correspond to the P—O fundamental vibrational modes, while the second region, spanning from 624 to 431 cm^−1^, indicates the bending modes of O—P—O. These two groups of bands exhibit similarities with those observed in the A_3_(PO_4_)_2_ family (Jin *et al.*, 2014 ▸). Specifically, the bands located at 1051, 1026, 971, and 936 cm^−1^ in β-Cd_3_(PO_4_)_2_ correspond to the fundamental vibrational modes of the symmetric P—O stretching, while the bands at 624, 597, 570, 558, 544, 531, and 431 cm^−1^ are assigned to the bending modes of O—P—O. Table 1 ▸ summarizes the bands and their corresponding assignments.

**Morphology of the powders**

**Figure 6:**
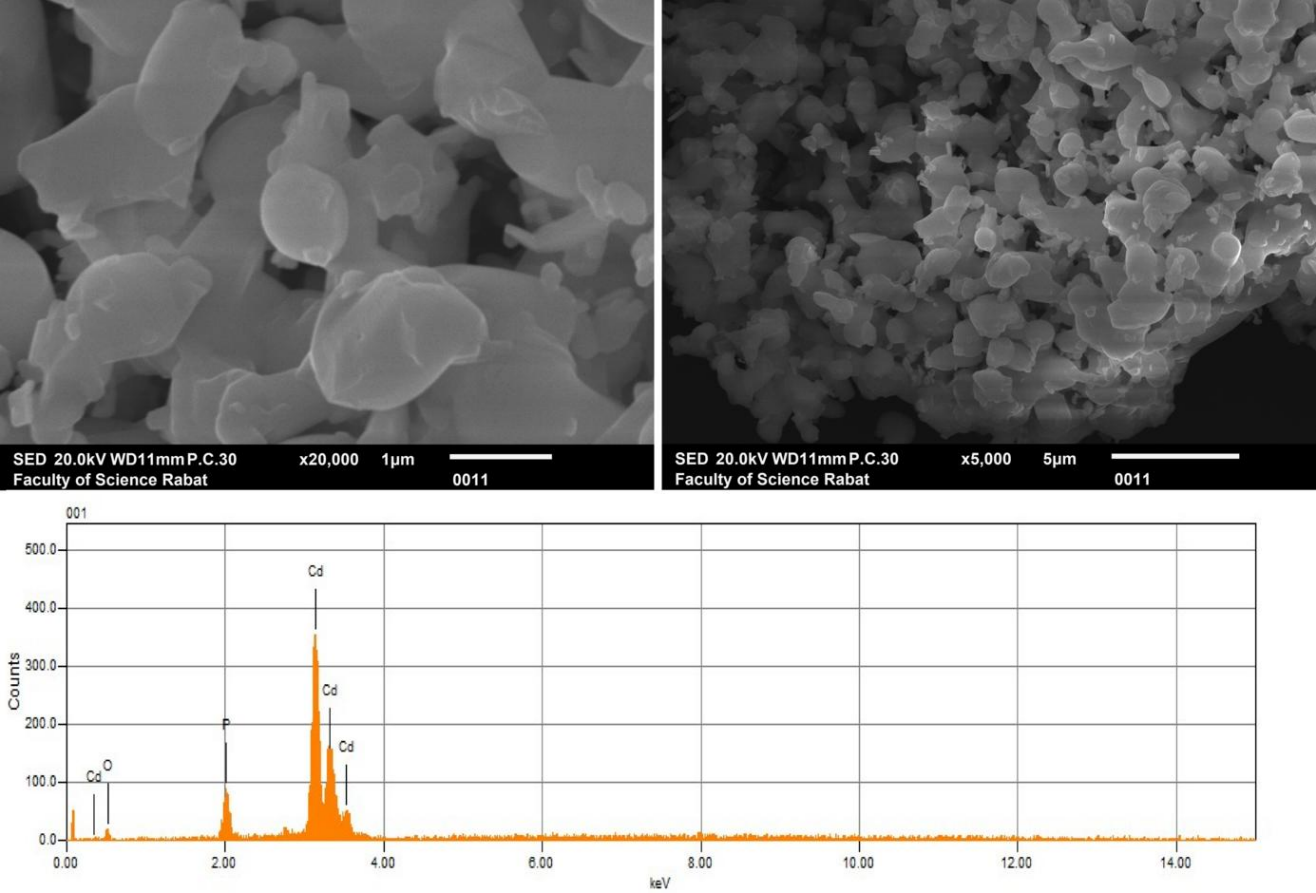
SEM images and EDX spectra of β-Cd_3_(PO_4_)_2_.

In Fig. 6 ▸, the morphology of β-Cd_3_(PO_4_)_2_ powders is depicted, showing particulate structures of a pulverized powder. The micrographic analysis indicates that the grains possess a well-defined shape. Furthermore, the EDX analysis confirms the purity and composition of the compound, which was also reported by Rajasri *et al.* (2019 ▸), thereby verifying its high quality.

**UV–Visible spectroscopy analysis.**

**Figure 7:**
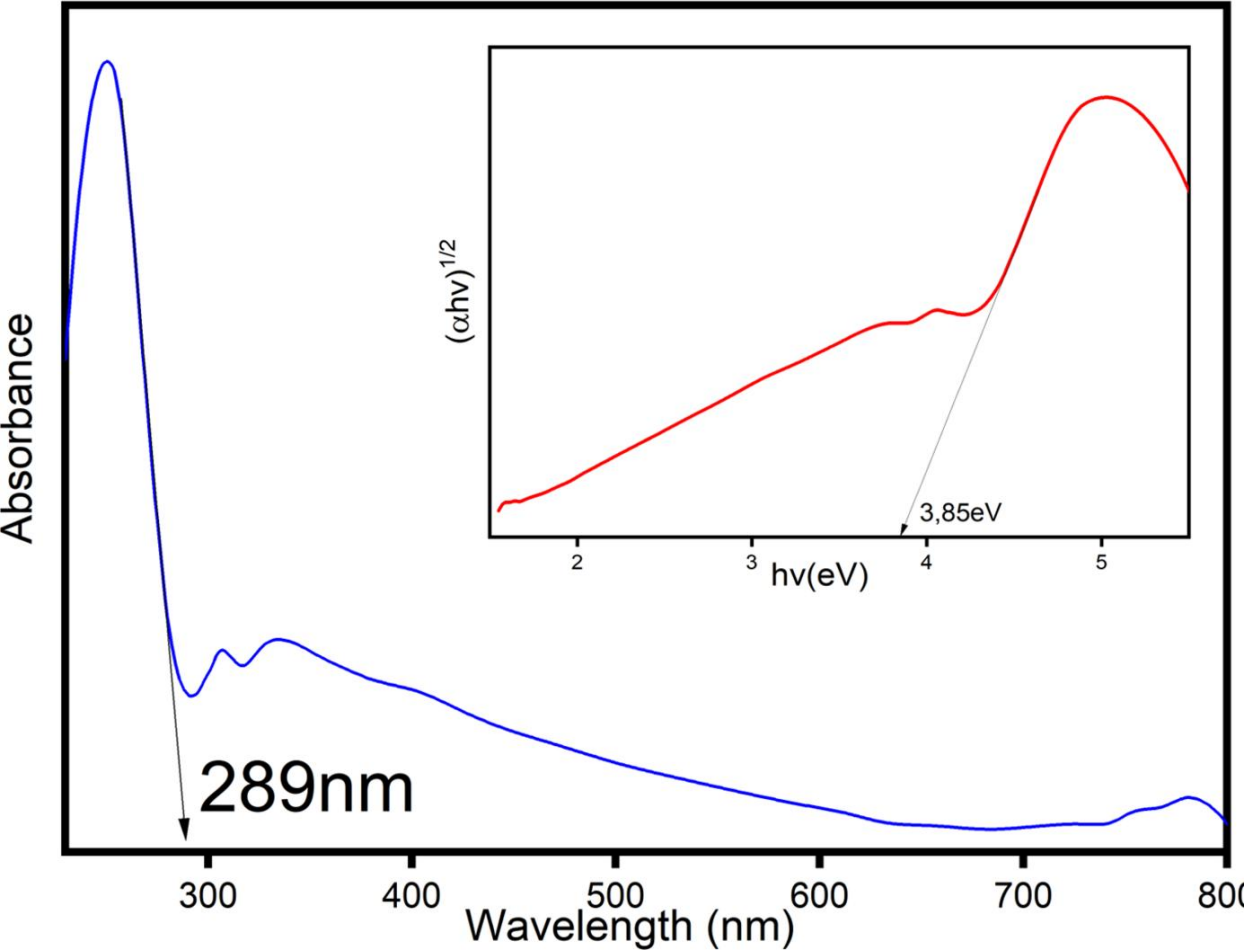
UV–Vis absorption spectra of β-Cd_3_(PO_4_)_2_. The inset shows the plot of (αhν)^1/2^ for determining the band-gap energy.

UV–Visible absorbance spectra of the β-Cd_3_(PO_4_)_2_ compound is presented in Fig. 7 ▸. The analysis was performed on a powder sample. An absorbance band is observed at 289 nm. The Kubelka–Munk analyses are required to determine the experimental band-gap energy. The band gap energy is the crossing point between the linear inclination of the absorption band and the energy axis. The estimated optical indirect band-gap energy is 3.85 eV. This energy value roughly places this phosphate in the class of semiconductors.

## 3. Database survey

The crystal structure of the tricadmium diorthophosphate, namely β-Cd_3_(PO_4_)_2_, was determined by Stephens (1967 ▸) using X-ray diffraction data collected from Weissenberg photographs. Its corresponding high-temperature form crystallizes in the monoclinic system and presents structural similarities with the β-Mn_3_(PO_4_)_2_ graftonite type (Stephens & Calvo, 1969 ▸). In light of this literature, β-Cd_3_(PO_4_)_2_ adopts the monoclinic space group *P*2_1_/c with the following cell parameters: *a* = 9.221 (1) Å, *b* = 10.335 (1) Å, *c* = 24.902 (5) Å, and β = 120.7 (2)° (Stephens, 1967 ▸; see Table 2 ▸). However, the crystal structure details are not readily available in the published articles. Furthermore, during our research on transition-metal-based phosphates, we have synthesized β-Cd_3_(PO_4_)_2_ crystals that crystallize in the monoclinic system with the lattice parameters *a*′ = 9.1895 Å, *b*′ = 10.3507 Å, *c*′ = 21.6887 Å, β′ = 99.64°, space group *P*2_1_/*n* (see Table 2 ▸). In fact, these parameters are related to those found by Stephens through the following basis transformation *a*′ = *a*, *b*′ = *b* and *c*′ = *a* + *c*. Although there is a relationship between the unit-cell parameters, it is very difficult to compare the two structural models due to the low quality of the Stephens (1967 ▸) model. Thus, we cannot conclude that it is the same structure.

It is important to note that the present structural model is obtained from the resolution and least-squares refinement of single crystal X-ray diffraction data (8280 reflections), measured with high precision. The low values of the reliability factors *R* and *Rw* (see Table 3 ▸) show that this model is correct. Moreover, the precisions of the inter­atomic distances and angles calculated from the atomic positions are very satisfactory and the values are compatible with the P—O and Cd—O distances and the O—P—O and O—Cd—O angles given in the literature of this type of phosphate. In the recent model, the cadmium–oxygen (Cd—O) bonds vary between 2.1454 and 2.5935 Å. In contrast, Stephens’ structure shows a more varied Cd—O bond length, ranging from 2.13 to 2.7 Å. Furthermore, the current structure portrays the phosphate tetra­hedra with regular geometries, wherein phospho­rus–oxygen (P—O) bond distances are consistently between 1.527 and 1.556 Å. Stephens’ model, on the other hand, presents a wider P—O bond distance variation, from 1.44 to 1.63 Å. The irregularities observed in the Stephens’ polyhedral units potentially stem from the aforementioned data resolution limitations of that study.

The rich crystal chemistry of the 3*d* transition-metal (II) orthophosphates attracts scientists to study their physico-chemical properties. From the crystallographic point of view, the most commonly adopted symmetry for the *M*_3_(PO_4_)_2_ family is the monoclinic system, space group *P*2_1_/*c*. Table 2 ▸ summarizes the crystallographic data for a selection of compounds belonging to this family. It appears from analysis of this table that the structural study of practically all phosphates belonging to this family has long been carried out, except Cr_3_(PO_4_)_2_. The latter phosphate crystal structure, constructed from CrO_5_, CrO_6_ and PO_4_ polyhedra, is closely related to the studied phosphate in the present work. However, a structural reinvestigation of some phosphates, such as Ni_3_(PO_4_)_2_ and Mn_3_(PO_4_)_2_, has been undertaken in recent years, as shown in Table 2 ▸.

## 4. Synthesis and crystallization

Single crystals of β-Cd_3_(PO_4_)_2_ were synthesized by a hydro­thermal process using the following protocol. In a Teflon beaker of 23 mL, cadmium nitrate (0.567 g, 99%) and phospho­ric acid (1.09 mL of a solution of 14.615 *M*) were mixed in the molar ratio Cd(NO_3_)_2_:H_3_PO_4_ = 3:2, and 12 mL of distilled water were added to the mix. The Teflon beaker was placed in the autoclave, carefully sealed, and heated at 473 K for two days. The resulting product constituted two single-crystal types with different shapes. Binocular observations allowed us to estimate the percentage of the two different crystal forms at 50% each. Single-crystal X-ray analysis revealed that the first one corresponds to the well-known compound Cd_5_(PO_4_)_3_OH (Hata *et al.*, 1978 ▸), a prism-shaped phosphate, while the second type, which is parallelepiped shaped, is the subject of the present work and was identified as β-Cd_3_(PO_4_)_2_.

The powder of the studied phosphate was synthesized by means of solid-state reaction carried out in air. Cadmium nitrate (99%), and di-ammonium hydrogen-phosphate (99%) were weighed at a molar ratio of 3:2 and ground thoroughly in an agate mortar. The mixture was pre-heated at 423 K, 623 K, and 823 K. The resulting powder was then ground thoroughly and heated to 1273 K for 24 h to obtain pure β-Cd_3_(PO_4_)_2_.

**Experimental details**

X-ray powder diffraction data were collected at room temperature using a Shimadzu diffractometer model LABXRD-6100, equipped with a secondary monochromator and Cu *K*α radiation (λ = 1.54056 Å). The X-ray diffraction data were collected at 40 kV in the inter­val 10° ≤ 2θ ≤ 70° with a step of 0.04 in 2θ and a counting time of 1.2 s per step. The collected XRD pattern was fitted using *JANA2006* software (Petříček *et al.*, 2014 ▸). The morphology and composition of the synthesized material were characterized using a JEOL JSM-IT 100 scanning electron microscope (SEM) equipped with an EDX at an accelerating voltage of 20 kV. Fourier-transform infrared spectroscopy (FTIR) was performed using a Bruker Platinum-ATR instrument. UV–Visible absorbance measurements were performed on powder samples using a JASCO instrument in the range of 190 to 900 nm at room temperature. The crystal structures were visualized using *DIAMOND* crystal and mol­ecular structure software (Bergerhoff *et al.*, 1996 ▸).

## 5. Refinement

Crystal data, data collection and structure refinement details are summarized in Table 3 ▸.

## Supplementary Material

Crystal structure: contains datablock(s) I. DOI: 10.1107/S2056989023009775/oo2001sup1.cif

Structure factors: contains datablock(s) I. DOI: 10.1107/S2056989023009775/oo2001Isup2.hkl

CCDC reference: 2306650

Additional supporting information:  crystallographic information; 3D view; checkCIF report

## Figures and Tables

**Table 1:** Infrared bands of β-Cd_3_(PO_4_)_2_ and their assignments

Band	Assignment
431	PO_4_ v2 out of plane bending modes
531	PO_4_ v4 out of plane bending modes
544	PO_4_ v4 out of plane bending modes
558	PO_4_ v4 out of plane bending modes
570	PO_4_ v4 out of plane bending modes
597	PO_4_ v4 out of plane bending modes
624	PO_4_ v4 out of plane bending modes
936	The symmetric P—O stretching corresponds to the undamental vibrational mode v1
971	The symmetric P—O stretching corresponds to the fundamental vibrational mode v1
1026	The triple-degenerate asymmetric P—O stretching mode corresponds to the v3 fundamental vibrational mode
1051	The triple-degenerate asymmetric P—O stretching mode corresponds to the v3 fundamental vibrational mode

**Table 2:** Divalent cation-based orthophosphates [*M*_3_(PO_4_)_2_] summary crystallographic data (Å, °, Å^3^)

Compound	Space group	*a*	*b*	*c*	β	*Z*	*V*	Reference
Ca_3_(PO_4_)_2_	*P*2_1_/*a*	12.89 (6)	27.28 (5)	15.22 (3)	126.2 (9)	24	4317.5	Mathew *et al.* (1977 ▸)
Cd_3_(PO_4_)_2_	*P*2_1_/*c*	9.22 (4)	10.34 (9)	24.90 (2)	120.7 (1)	12	2030.0	Stephens (1967 ▸)
	*P*2_1_/*n*	9.19 (7)	10.35 (1)	21.69 (9)	99.6 (2)	12	2033.8	Present work
Co_3_(PO_4_)_2_	*P*2_1_/*n*	5.06 (8)	8.36 (2)	8.79 (4)	121.0 (1)	2	319.4	Anderson *et al.* (1975 ▸)
Cr_3_(PO_4_)_2_	*P*2_1_/*n*	4.97 (9)	9.50 (3)	6.48 (2)	91.4 (3)	2	305.6	Glaum *et al.* (2011 ▸)
Fe_3_(PO_4_)_2_	*P*2_1_/*a*	10.44 (3)	4.79 (2)	6.03 (2)	91.0 (5)	2	301.3	Ericsson & Khangi (1988 ▸)
	*P*2_1_/*n*	8.88 (2)	11.17 (3)	6.15 (8)	99.4 (8)	4	601.0	Kostiner & Rea (1974 ▸)
Mg_3_(PO_4_)_2_	*P*2_1_/*n*	7.60 (7)	8.23 (1)	5.08 (1)	94.1 (5)	2	316.6	Nord & Kierkegaard (1968 ▸)
	*P*21/*m*	7.605 (2)	8.233 (3)	5.080 (1)	94.19 (3)	2	317.2	Baykal *et al.* (1997 ▸)
	*P*2_1_/*n*	10.25 (9)	4.72 (2)	5.92 (4)	90.9 (1)	2	287.0	Nord & Stefanidis (1983 ▸)
Mn_3_(PO_4_)_2_	*P*2_1_/*c*	8.94 (3)	10.04 (1)	24.12 (8)	120.8 (3)	12	1861.1	Stephens & Calvo (1969 ▸)
	*P*2_1_/*c*	8.80 (4)	11.45 (1)	6.25 (5)	99.0 (2)	4	621.9	Volkova *et al.* (2016 ▸)
	*P*2_1_/*c*	8.92 (1)	9.15 (9)	8.66 (9)	111.7 (1)	4	657.2	Neher & Salguero (2017 ▸)
Ni_3_(PO_4_)_2_	*P*2_1_/*n*	5.82 (6)	4.69 (2)	10.10 (5)	91.1 (3)	2	276.1	Escobal *et al.* (2005 ▸)
	*P*2_1_/*c*	8.70 (2)	11.12 (1)	6.11 (2)	100.0 (8)	4	581.7	Nord & Stefanidis (1983 ▸)
Pb_3_(PO_4_)_2_	*C*2/*c*	13.81 (8)	5.69 (8)	9.43 (3)	102.4 (3)	4	723.5	Brixner *et al.* (1973 ▸)
Sr_3_(PO_4_)_2_	*R* 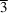 *m*	5.3901 (8)	5.3901 (8)	19.785 (5)		4	497.8	Sugiyama & Tokonami (1990 ▸)
Ba_3_(PO_4_)_2_	*R* 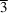 *m*	5.6038 (7)	5.6038 (7)	21.000 (5)		4	571.1	Sugiyama & Tokonami (1990 ▸)
Zn_3_(PO_4_)_2_	*P*2_1_/*c*	5.07 (2)	8.47 (3)	8.77 (2)	120.5 (5)	2	323.1	Calvo (1963 ▸); Stephens & Calvo (1967 ▸)
	*C*2/*c*	8.14 (7)	5.63 (3)	15.04 (9)	105.1 (8)	4	665.4	Calvo (1965 ▸)
	*P*2_1/_ *n*	9.39 (8)	9.17 (1)	8.69 (3)	125.7 (3)	4	607.3	Stephens & Calvo (1969 ▸)

**Table 3:** Experimental details

Crystal data	
Chemical formula	Cd_3_(PO_4_)_2_
*M*_r_	527.14
Crystal system, space group	Monoclinic, *P*2_1_/*n*
Temperature (K)	296
*a*, *b*, *c* (Å)	9.1895 (7), 10.3507 (8), 21.6887 (16)
β (°)	99.644 (3)
*V* (Å^3^)	2033.8 (3)
*Z*	12
Radiation type	Mo *K*α
μ (mm^−1^)	9.81
Crystal size (mm)	0.31 × 0.27 × 0.22

Data collection	
Diffractometer	Bruker X8 *APEX* Diffractometer
Absorption correction	Multi-scan (*SADABS*; Krause *et al.*, 2015 ▸)
*T*_min_, *T*_max_	0.544, 0.747
No. of measured, independent and observed [*I* > 2σ(*I*)] reflections	129694, 9851, 8280
*R*_int_	0.047
(sin θ/λ)_max_ (Å^−1^)	0.833

Refinement	
*R*[*F* ^2^ > 2σ(*F* ^2^)], *wR*(*F* ^2^), *S*	0.023, 0.054, 1.07
No. of reflections	9851
No. of parameters	352
Δρ_max_, Δρ_min_ (e Å^−3^)	1.60, −1.74

Computer programs: *APEX3* and *SAINT* (Bruker, 2016 ▸), *SHELXT2014/5* (Sheldrick, 2015*a* ▸), *SHELXL2018/3* (Sheldrick, 2015*b* ▸), *ORTEP-3 for Windows* (Farrugia, 2012 ▸), *DIAMOND* (Brandenburg, 2006 ▸) and *publCIF* (Westrip, 2010 ▸).

## supplementary crystallographic information

### Crystal data

**Table:**

Cd_3_(PO_4_)_2_	*F*(000) = 2856
*M**_r_* = 527.14	*D*_x_ = 5.165 Mg m^−^^3^
Monoclinic, *P*2_1_/*n*	Mo *K*α radiation, λ = 0.71073 Å
*a* = 9.1895 (7) Å	Cell parameters from 9851 reflections
*b* = 10.3507 (8) Å	θ = 2.2–36.3°
*c* = 21.6887 (16) Å	µ = 9.81 mm^−^^1^
β = 99.644 (3)°	*T* = 296 K
*V* = 2033.8 (3) Å^3^	Parallelepiped, colourless
*Z* = 12	0.31 × 0.27 × 0.22 mm

### Data collection

**Table:**

Bruker X8 APEX Diffractometer	9851 independent reflections
Radiation source: fine-focus sealed tube	8280 reflections with *I* > 2σ(*I*)
Graphite monochromator	*R*_int_ = 0.047
φ and ω scans	θ_max_ = 36.3°, θ_min_ = 2.2°
Absorption correction: multi-scan (SADABS; Krause *et al.*, 2015)	*h* = −15→15
*T*_min_ = 0.544, *T*_max_ = 0.747	*k* = −17→17
129694 measured reflections	*l* = −36→36

### Refinement

**Table:**

Refinement on *F*^2^	0 restraints
Least-squares matrix: full	Primary atom site location: dual
*R*[*F*^2^ > 2σ(*F*^2^)] = 0.023	Secondary atom site location: difference Fourier map
*wR*(*F*^2^) = 0.054	*w* = 1/[σ^2^(*F*_o_^2^) + (0.0205*P*)^2^ + 4.3579*P*] where *P* = (*F*_o_^2^ + 2*F*_c_^2^)/3
*S* = 1.07	(Δ/σ)_max_ = 0.004
9851 reflections	Δρ_max_ = 1.60 e Å^−^^3^
352 parameters	Δρ_min_ = −1.74 e Å^−^^3^

### Special details

**Table:**

Geometry. All esds (except the esd in the dihedral angle between two l.s. planes) are estimated using the full covariance matrix. The cell esds are taken into account individually in the estimation of esds in distances, angles and torsion angles; correlations between esds in cell parameters are only used when they are defined by crystal symmetry. An approximate (isotropic) treatment of cell esds is used for estimating esds involving l.s. planes.
Refinement. The crystal structure of β-Cd_3_(PO_4_)_2_ was investigated using single-crystal X-ray diffraction data collected at room temperature with a Bruker D8 Venture Super DUO Diffractometer equipped with a PHOTON100 CMOS area-detector and monochromatic MoKα radiation (λ=0.71073 Å). APEX3 (Bruker, APEX3 (Version 5.054), SAINT (Version 6.36A), SADABS. (Bruker, 2016) software was used for data reduction and the absorption correction was performed by multi-scan semi-empirical method using SADABS program (Krause, *et al.* 2015). The crystal structure was solved using dual space algorithm as implemented in SHELXT program (Sheldrick, 2015*a*), completed by a Difference Fourier map and refined by least-squares using SHELXL program (Sheldrick 2015*b*) integrated into the WinGX interface (Farrugia, 2012).

### Fractional atomic coordinates and isotropic or equivalent isotropic displacement parameters (Å^2^)

**Table:**

	*x*	*y*	*z*	*U*_iso_*/*U*_eq_	
Cd1	0.74691 (2)	0.71080 (2)	0.36476 (2)	0.01178 (3)	
Cd2	0.27909 (2)	0.39633 (2)	0.92293 (2)	0.01251 (3)	
Cd3	0.60228 (2)	0.39417 (2)	0.58439 (2)	0.01124 (3)	
Cd4	0.94569 (2)	0.28838 (2)	0.95686 (2)	0.01149 (3)	
Cd5	0.71202 (2)	0.41020 (2)	0.77990 (2)	0.01002 (3)	
Cd6	0.05148 (2)	0.60385 (2)	0.74786 (2)	0.01157 (3)	
Cd7	0.60491 (2)	0.60229 (2)	0.89221 (2)	0.00988 (3)	
Cd8	0.40399 (2)	0.70425 (2)	0.70409 (2)	0.01088 (3)	
Cd9	0.93970 (2)	0.59251 (2)	0.56014 (2)	0.01049 (3)	
P1	0.69824 (6)	0.43486 (5)	0.43995 (2)	0.00739 (9)	
P2	0.95135 (6)	0.36168 (6)	0.67096 (3)	0.00825 (9)	
P3	0.69971 (7)	0.64250 (6)	0.66858 (3)	0.00801 (9)	
P4	0.35072 (6)	0.42713 (5)	0.77938 (2)	0.00705 (9)	
P5	−0.03383 (6)	0.57903 (5)	0.89456 (2)	0.00714 (9)	
P6	0.62768 (7)	0.35381 (6)	1.00079 (3)	0.00835 (9)	
O1	0.7195 (2)	0.32411 (17)	0.39518 (8)	0.0153 (3)	
O2	0.62835 (19)	0.55464 (17)	0.40326 (8)	0.0120 (3)	
O3	0.84808 (19)	0.46875 (17)	0.47901 (8)	0.0132 (3)	
O4	0.5870 (2)	0.39020 (19)	0.48128 (8)	0.0158 (3)	
O5	0.8179 (2)	0.30534 (18)	0.62739 (9)	0.0161 (3)	
O6	1.0338 (2)	0.4652 (2)	0.63983 (10)	0.0255 (5)	
O7	1.0579 (2)	0.24766 (18)	0.68907 (8)	0.0166 (3)	
O8	0.9105 (2)	0.41803 (19)	0.73139 (8)	0.0175 (4)	
O9	0.7346 (2)	0.57633 (18)	0.60939 (8)	0.0153 (3)	
O10	0.5947 (2)	0.75733 (17)	0.64955 (8)	0.0149 (3)	
O11	0.8400 (2)	0.70048 (17)	0.70769 (9)	0.0154 (3)	
O12	0.6177 (2)	0.5506 (2)	0.70596 (9)	0.0204 (4)	
O13	0.28310 (19)	0.54515 (17)	0.74156 (8)	0.0117 (3)	
O14	0.3730 (2)	0.31593 (17)	0.73519 (8)	0.0138 (3)	
O15	0.50013 (19)	0.45790 (17)	0.82041 (8)	0.0125 (3)	
O16	0.2369 (2)	0.38617 (18)	0.82065 (8)	0.0138 (3)	
O17	0.0602 (2)	0.63773 (19)	0.84925 (8)	0.0163 (3)	
O18	−0.18533 (19)	0.53473 (17)	0.85947 (8)	0.0128 (3)	
O19	−0.0598 (2)	0.68256 (17)	0.94219 (8)	0.0136 (3)	
O20	0.05089 (19)	0.46386 (16)	0.92864 (8)	0.0117 (3)	
O21	0.4881 (2)	0.30043 (18)	0.95967 (9)	0.0168 (3)	
O22	0.6649 (2)	0.49060 (17)	0.98153 (8)	0.0156 (3)	
O23	0.7475 (2)	0.25479 (18)	0.99447 (9)	0.0186 (4)	
O24	0.6119 (2)	0.36004 (19)	1.07107 (8)	0.0167 (3)	

### Atomic displacement parameters (Å^2^)

**Table:**

	*U*^11^	*U*^22^	*U*^33^	*U*^12^	*U*^13^	*U*^23^
Cd1	0.01169 (7)	0.01256 (7)	0.01087 (6)	−0.00280 (6)	0.00125 (5)	0.00014 (5)
Cd2	0.01060 (7)	0.01629 (8)	0.00983 (6)	0.00050 (6)	−0.00060 (5)	−0.00269 (5)
Cd3	0.00935 (7)	0.01100 (7)	0.01280 (7)	−0.00021 (5)	0.00021 (5)	−0.00116 (5)
Cd4	0.01258 (7)	0.01305 (7)	0.00873 (6)	−0.00046 (6)	0.00142 (5)	0.00123 (5)
Cd5	0.01194 (7)	0.00914 (7)	0.00902 (6)	−0.00021 (5)	0.00187 (5)	0.00066 (5)
Cd6	0.00914 (7)	0.01060 (7)	0.01430 (7)	−0.00063 (5)	0.00009 (5)	−0.00122 (5)
Cd7	0.01032 (7)	0.01008 (7)	0.00935 (6)	0.00014 (5)	0.00200 (5)	0.00078 (5)
Cd8	0.01204 (7)	0.01262 (7)	0.00817 (6)	0.00067 (5)	0.00220 (5)	0.00167 (5)
Cd9	0.01215 (7)	0.01086 (7)	0.00841 (6)	0.00036 (5)	0.00158 (5)	0.00089 (5)
P1	0.0078 (2)	0.0074 (2)	0.0064 (2)	−0.00011 (18)	−0.00040 (17)	−0.00001 (17)
P2	0.0080 (2)	0.0090 (2)	0.0074 (2)	0.00023 (18)	0.00032 (17)	−0.00032 (18)
P3	0.0085 (2)	0.0079 (2)	0.0072 (2)	0.00045 (18)	0.00022 (17)	−0.00030 (17)
P4	0.0076 (2)	0.0069 (2)	0.00626 (19)	−0.00028 (18)	0.00013 (17)	−0.00019 (17)
P5	0.0077 (2)	0.0068 (2)	0.0065 (2)	−0.00028 (18)	−0.00001 (17)	−0.00010 (17)
P6	0.0090 (2)	0.0093 (2)	0.0066 (2)	−0.00031 (18)	0.00063 (18)	0.00027 (18)
O1	0.0210 (9)	0.0105 (7)	0.0122 (7)	0.0037 (6)	−0.0040 (6)	−0.0047 (6)
O2	0.0108 (7)	0.0113 (7)	0.0139 (7)	0.0031 (6)	0.0018 (6)	0.0041 (6)
O3	0.0094 (7)	0.0163 (8)	0.0127 (7)	−0.0006 (6)	−0.0016 (6)	−0.0052 (6)
O4	0.0163 (8)	0.0198 (9)	0.0118 (7)	−0.0071 (7)	0.0042 (6)	0.0019 (6)
O5	0.0124 (8)	0.0132 (8)	0.0194 (8)	0.0010 (6)	−0.0070 (6)	−0.0054 (6)
O6	0.0160 (9)	0.0360 (12)	0.0225 (9)	−0.0078 (8)	−0.0023 (7)	0.0188 (9)
O7	0.0176 (9)	0.0154 (8)	0.0157 (8)	0.0064 (7)	−0.0010 (6)	−0.0020 (6)
O8	0.0161 (9)	0.0231 (9)	0.0145 (7)	−0.0024 (7)	0.0060 (6)	−0.0087 (7)
O9	0.0155 (8)	0.0166 (8)	0.0145 (7)	−0.0019 (7)	0.0048 (6)	−0.0060 (6)
O10	0.0162 (8)	0.0117 (8)	0.0157 (7)	0.0051 (6)	−0.0004 (6)	0.0013 (6)
O11	0.0124 (8)	0.0102 (8)	0.0203 (8)	0.0006 (6)	−0.0066 (6)	−0.0037 (6)
O12	0.0143 (9)	0.0254 (10)	0.0212 (9)	−0.0013 (7)	0.0022 (7)	0.0144 (8)
O13	0.0094 (7)	0.0108 (7)	0.0144 (7)	0.0015 (6)	0.0002 (6)	0.0046 (6)
O14	0.0175 (8)	0.0109 (7)	0.0116 (7)	0.0043 (6)	−0.0020 (6)	−0.0042 (6)
O15	0.0095 (7)	0.0145 (8)	0.0122 (7)	0.0004 (6)	−0.0019 (6)	−0.0030 (6)
O16	0.0138 (8)	0.0177 (8)	0.0103 (7)	−0.0043 (6)	0.0034 (6)	0.0015 (6)
O17	0.0179 (9)	0.0194 (9)	0.0122 (7)	−0.0069 (7)	0.0045 (6)	0.0041 (6)
O18	0.0080 (7)	0.0155 (8)	0.0141 (7)	−0.0006 (6)	−0.0004 (6)	−0.0058 (6)
O19	0.0199 (9)	0.0095 (7)	0.0098 (6)	0.0042 (6)	−0.0025 (6)	−0.0026 (6)
O20	0.0115 (7)	0.0093 (7)	0.0141 (7)	0.0022 (6)	0.0015 (6)	0.0029 (6)
O21	0.0134 (8)	0.0153 (8)	0.0185 (8)	−0.0015 (6)	−0.0065 (6)	−0.0026 (7)
O22	0.0207 (9)	0.0112 (8)	0.0133 (7)	−0.0040 (6)	−0.0017 (6)	0.0036 (6)
O23	0.0153 (9)	0.0152 (8)	0.0272 (9)	0.0027 (7)	0.0092 (7)	−0.0004 (7)
O24	0.0165 (9)	0.0258 (10)	0.0080 (6)	0.0053 (7)	0.0030 (6)	0.0029 (6)

### Geometric parameters (Å, º)

**Table:**

Cd1—O2	2.1913 (17)	Cd8—O13	2.2156 (17)
Cd1—O14^i^	2.2782 (17)	Cd8—O1^i^	2.2783 (17)
Cd1—O17^ii^	2.3068 (18)	Cd8—O16^xii^	2.2973 (18)
Cd1—O24^iii^	2.3243 (18)	Cd8—O7^iii^	2.3285 (18)
Cd1—O7^iv^	2.338 (2)	Cd8—O10	2.3381 (19)
Cd2—O16	2.1896 (17)	Cd8—O12	2.522 (2)
Cd2—O21	2.1900 (19)	Cd9—O3	2.2248 (17)
Cd2—O20	2.2336 (18)	Cd9—O6	2.2297 (19)
Cd2—O22^v^	2.3602 (18)	Cd9—O21^iii^	2.3135 (19)
Cd2—O1^vi^	2.3996 (18)	Cd9—O9	2.3230 (18)
Cd3—O4	2.2176 (17)	Cd9—O3^iv^	2.3411 (18)
Cd3—O5	2.2409 (18)	Cd9—O23^iii^	2.550 (2)
Cd3—O2^i^	2.2437 (17)	P1—O3	1.5316 (18)
Cd3—O9	2.2598 (18)	P1—O1	1.5358 (18)
Cd3—O19^vii^	2.2818 (18)	P1—O4	1.5388 (18)
Cd4—O23	2.1454 (19)	P1—O2	1.5529 (17)
Cd4—O20^viii^	2.1924 (17)	P2—O6	1.533 (2)
Cd4—O4^ix^	2.2702 (18)	P2—O5	1.5327 (18)
Cd4—O19^v^	2.2836 (17)	P2—O8	1.5376 (18)
Cd4—O10^x^	2.2978 (18)	P2—O7	1.5417 (19)
Cd5—O12	2.2292 (18)	P3—O12	1.5283 (19)
Cd5—O18^viii^	2.2326 (17)	P3—O9	1.5354 (18)
Cd5—O11^x^	2.2486 (18)	P3—O11	1.5409 (18)
Cd5—O8	2.2543 (18)	P3—O10	1.5430 (18)
Cd5—O15	2.3196 (18)	P4—O14	1.5334 (18)
Cd6—O17	2.2150 (18)	P4—O15	1.5391 (18)
Cd6—O11^xi^	2.2293 (18)	P4—O13	1.5430 (17)
Cd6—O13	2.2396 (17)	P4—O16	1.5451 (18)
Cd6—O14^xii^	2.3131 (18)	P5—O19	1.5348 (18)
Cd6—O8^xi^	2.313 (2)	P5—O17	1.5383 (18)
Cd7—O22	2.2441 (17)	P5—O18	1.5404 (18)
Cd7—O15	2.2547 (17)	P5—O20	1.5429 (17)
Cd7—O18^viii^	2.2732 (17)	P6—O23	1.527 (2)
Cd7—O5^iii^	2.2808 (18)	P6—O22	1.5311 (19)
Cd7—O24^v^	2.2990 (19)	P6—O21	1.5372 (19)
Cd7—O7^iii^	2.5935 (19)	P6—O24	1.5566 (18)
			
O2—Cd1—O14^i^	94.64 (7)	O15—Cd7—O7^iii^	77.53 (6)
O2—Cd1—O17^ii^	98.73 (7)	O18^viii^—Cd7—O7^iii^	112.89 (6)
O14^i^—Cd1—O17^ii^	73.41 (6)	O5^iii^—Cd7—O7^iii^	60.05 (6)
O2—Cd1—O24^iii^	121.70 (6)	O24^v^—Cd7—O7^iii^	72.69 (6)
O14^i^—Cd1—O24^iii^	141.54 (7)	O13—Cd8—O1^i^	92.86 (7)
O17^ii^—Cd1—O24^iii^	87.86 (7)	O13—Cd8—O16^xii^	113.40 (7)
O2—Cd1—O7^iv^	142.93 (6)	O1^i^—Cd8—O16^xii^	73.32 (6)
O14^i^—Cd1—O7^iv^	80.58 (7)	O13—Cd8—O7^iii^	77.26 (6)
O17^ii^—Cd1—O7^iv^	114.60 (7)	O1^i^—Cd8—O7^iii^	158.96 (7)
O24^iii^—Cd1—O7^iv^	77.21 (6)	O16^xii^—Cd8—O7^iii^	93.39 (6)
O16—Cd2—O21	110.02 (7)	O13—Cd8—O10	145.55 (6)
O16—Cd2—O20	93.60 (7)	O1^i^—Cd8—O10	81.31 (7)
O21—Cd2—O20	153.63 (7)	O16^xii^—Cd8—O10	97.48 (6)
O16—Cd2—O22^v^	152.72 (7)	O7^iii^—Cd8—O10	117.23 (7)
O21—Cd2—O22^v^	81.75 (7)	O13—Cd8—O12	87.84 (6)
O20—Cd2—O22^v^	82.22 (7)	O1^i^—Cd8—O12	101.36 (7)
O16—Cd2—O1^vi^	72.91 (6)	O16^xii^—Cd8—O12	158.06 (6)
O21—Cd2—O1^vi^	79.03 (7)	O7^iii^—Cd8—O12	96.82 (7)
O20—Cd2—O1^vi^	97.73 (7)	O10—Cd8—O12	60.58 (6)
O22^v^—Cd2—O1^vi^	134.32 (6)	O3—Cd9—O6	108.60 (8)
O4—Cd3—O5	108.31 (7)	O3—Cd9—O21^iii^	118.11 (7)
O4—Cd3—O2^i^	102.91 (7)	O6—Cd9—O21^iii^	127.43 (7)
O5—Cd3—O2^i^	146.53 (7)	O3—Cd9—O9	95.37 (6)
O4—Cd3—O9	101.42 (7)	O6—Cd9—O9	80.79 (7)
O5—Cd3—O9	80.78 (7)	O21^iii^—Cd9—O9	115.63 (7)
O2^i^—Cd3—O9	104.80 (7)	O3—Cd9—O3^iv^	77.59 (7)
O4—Cd3—O19^vii^	75.41 (6)	O6—Cd9—O3^iv^	83.26 (7)
O5—Cd3—O19^vii^	79.23 (6)	O21^iii^—Cd9—O3^iv^	84.39 (7)
O2^i^—Cd3—O19^vii^	97.58 (7)	O9—Cd9—O3^iv^	159.49 (7)
O9—Cd3—O19^vii^	157.50 (7)	O3—Cd9—O23^iii^	82.85 (6)
O23—Cd4—O20^viii^	133.04 (7)	O6—Cd9—O23^iii^	153.31 (7)
O23—Cd4—O4^ix^	105.57 (7)	O21^iii^—Cd9—O23^iii^	59.54 (6)
O20^viii^—Cd4—O4^ix^	118.55 (7)	O9—Cd9—O23^iii^	74.04 (6)
O23—Cd4—O19^v^	86.54 (7)	O3^iv^—Cd9—O23^iii^	123.24 (6)
O20^viii^—Cd4—O19^v^	90.31 (7)	O3—P1—O1	108.84 (10)
O4^ix^—Cd4—O19^v^	74.37 (6)	O3—P1—O4	111.71 (10)
O23—Cd4—O10^x^	110.78 (8)	O1—P1—O4	108.13 (11)
O20^viii^—Cd4—O10^x^	80.80 (6)	O3—P1—O2	110.91 (10)
O4^ix^—Cd4—O10^x^	96.33 (6)	O1—P1—O2	110.94 (10)
O19^v^—Cd4—O10^x^	162.23 (7)	O4—P1—O2	106.28 (10)
O12—Cd5—O18^viii^	104.06 (7)	O6—P2—O5	113.44 (11)
O12—Cd5—O11^x^	130.59 (7)	O6—P2—O8	108.65 (12)
O18^viii^—Cd5—O11^x^	122.38 (7)	O5—P2—O8	112.67 (11)
O12—Cd5—O8	83.60 (7)	O6—P2—O7	107.92 (12)
O18^viii^—Cd5—O8	94.01 (7)	O5—P2—O7	105.92 (10)
O11^x^—Cd5—O8	107.18 (7)	O8—P2—O7	107.98 (11)
O12—Cd5—O15	83.32 (7)	O12—P3—O9	110.88 (12)
O18^viii^—Cd5—O15	81.78 (6)	O12—P3—O11	113.07 (11)
O11^x^—Cd5—O15	87.22 (7)	O9—P3—O11	111.18 (11)
O8—Cd5—O15	164.84 (7)	O12—P3—O10	106.22 (11)
O17—Cd6—O11^xi^	101.60 (7)	O9—P3—O10	109.20 (10)
O17—Cd6—O13	103.11 (7)	O11—P3—O10	105.98 (10)
O11^xi^—Cd6—O13	150.83 (7)	O14—P4—O15	108.48 (10)
O17—Cd6—O14^xii^	74.45 (6)	O14—P4—O13	110.24 (10)
O11^xi^—Cd6—O14^xii^	81.67 (6)	O15—P4—O13	112.60 (10)
O13—Cd6—O14^xii^	90.24 (7)	O14—P4—O16	109.29 (11)
O17—Cd6—O8^xi^	102.19 (6)	O15—P4—O16	110.29 (10)
O11^xi^—Cd6—O8^xi^	83.31 (7)	O13—P4—O16	105.89 (10)
O13—Cd6—O8^xi^	106.17 (7)	O19—P5—O17	108.76 (11)
O14^xii^—Cd6—O8^xi^	163.55 (7)	O19—P5—O18	108.10 (10)
O22—Cd7—O15	105.64 (7)	O17—P5—O18	111.09 (10)
O22—Cd7—O18^viii^	90.72 (7)	O19—P5—O20	109.87 (9)
O15—Cd7—O18^viii^	82.34 (6)	O17—P5—O20	108.60 (11)
O22—Cd7—O5^iii^	126.62 (7)	O18—P5—O20	110.39 (10)
O15—Cd7—O5^iii^	126.31 (7)	O23—P6—O22	113.50 (11)
O18^viii^—Cd7—O5^iii^	85.34 (7)	O23—P6—O21	104.52 (11)
O22—Cd7—O24^v^	83.70 (7)	O22—P6—O21	112.15 (10)
O15—Cd7—O24^v^	93.27 (7)	O23—P6—O24	107.62 (11)
O18^viii^—Cd7—O24^v^	171.73 (7)	O22—P6—O24	106.90 (11)
O5^iii^—Cd7—O24^v^	102.88 (7)	O21—P6—O24	112.17 (11)
O22—Cd7—O7^iii^	156.36 (7)		

Symmetry codes: (i) −*x*+1, −*y*+1, −*z*+1; (ii) *x*+1/2, −*y*+3/2, *z*−1/2; (iii) −*x*+3/2, *y*+1/2, −*z*+3/2; (iv) −*x*+2, −*y*+1, −*z*+1; (v) −*x*+1, −*y*+1, −*z*+2; (vi) *x*−1/2, −*y*+1/2, *z*+1/2; (vii) −*x*+1/2, *y*−1/2, −*z*+3/2; (viii) *x*+1, *y*, *z*; (ix) *x*+1/2, −*y*+1/2, *z*+1/2; (x) −*x*+3/2, *y*−1/2, −*z*+3/2; (xi) *x*−1, *y*, *z*; (xii) −*x*+1/2, *y*+1/2, −*z*+3/2.
